# Obstetrics

**Published:** 1859-01

**Authors:** 


### OBSTETRICS.

1. ON ALOES ENEMATA IN UTERINE CATARRH. By *Professor Aran.*—Of the numerous discharges which take place from the genitals, some have their seat in the vulva and lower part of the vagina, others are furnished by the upper parts of the vagina and by the mucous membrane of the cervix uteri, and others again proceed from the interior of the uterus. This is not a mere anatomical distinction; for the discharges having different seats are far from resembling each other, either in regard to their causes or the resistance they offer to treatment. The vulvo-vaginal discharges are usually connected with a perpetic influence, but they sometimes depend, especially in the young, upon the presence of ascarides in the rectum or vagina, or upon the practice of bad habits. Kept up by the friction of the opposite surfaces of the vulva and vagina, they are rapidly relieved when the irritated surfaces are kept separated from each other, and dusted with some inert powder.

The discharges which proceed from the upper portion of the vagina and the os uteri are generally connected with congestion, irritation, or inflammation of the uterus or neighboring parts. If we subdue such irritation, the discharge will subside of itself without any special treatment. The discharges furnished by the interior of the uterus are far from being of this simple character; and, by the admission of all practitioners, what has been termed *uterine catarrh* constitutes, in its chronic form, one of the most obstinate, not to say incurable, affections that can affect the uterine system.

According to the particular point furnishing the secretion, the catarrh exhibits very different appearances. Sometimes it is a liquid as transparent as water, but slightly viscid; sometimes it is a highly viscid, gelatinous, albuminous, transparent or opake mucus, streaked with white, gray, or yellow; and sometimes, again, it approaches more or less in its character to those of purulent secretions. These differences have been noted by all who have employed the speculum, but no one has pointed out the precise point at which these various secretions take place. M. Aran's numerous observations have shown him that the highly viscous, albuminous mucus always proceeds from the cavity of the cervix; and on the dead body we can trace into the crypts of the cervix numerous filaments, which united together form the large flakes, resembling white of egg, which are spread generally over the lower lip of the cervix, and thence into the vagina. The aqueous mucus, whether clear, sanguinolent, or even puriform, is furnished by the cavity of the body of the uterus. These two kinds of mucus present the characteristic peculiarity of being alkaline, while that furnished by the vagina and os uteri is always acid. Becoming mixed with the natural or increased secretion of the vagina, the complex discharges known as leucorrhœa are produced, consisting of more or less viscous secretions, according as they contain more or less of the mucus of the cervix, and more or less of a white or yellow color, according to their combination with the epithelial secretion of the vagina, or with pus derived from this canal or from the cavities of the uterus. Leucorrhœa whenever it is abundant is never furnished exclusively by the uterus, the secretion, from whatever part of its cavity it proceeds, being always slight. The secretion may take place regularly at certain hours in some patients, who are apprised of its occurrence by colicky pains; but it is never in sufficient quantity to give rise to the true "whites." It thus must be admitted that abnormal secretion of any importance cannot take place in the uterus without being followed by increased vaginal secretion; whether this arises from simple consensus, or whether the mucus secreted by the uterus directly stimulates the vaginal mucous membrane into increased action.

Every one acquainted with this affection is aware of its obstinacy, especially when it has continued for some time. Doubtless, in many cases, the resistance to treatment is explained by the presence of some lesion of the uterine appendages. An oophoritis, a chronic inflammation of the tube, or a partial peritonitis may too frequently keep up the irritation which maintains the catarrh; but in other cases, in which there is no reason to suspect such complication, the discharge is no less obstinate, and relapses with such facility as to drive both patient and practitioner to despair. Chance has of late years led M. Aran to the discovery of the means of curing, if not all, at least a great number of these cases, although he is far from wishing to substitute it for all other measures heretofore proposed—too many resources in so obstinate a disease not being in fact possible. After trying in vain to cure an obstinate case of amenorrhœa with chloro-anæmia, he determined to put into force Schonlein's treatment, which consists in the repeated administration of small enemata, composed of two and a half drachms of aloes to an ounce of mucilage. The amenorrhœa did not yield to this, but an abundant leucorrhœa, to which the patient had been subject for several years, entirely disappeared. His attention having been struck by this fact, he tried the same remedy under various circumstances; and the practice he now approves of is to administer (every day, or every other, according to the effect produced) first an enema of mere tepid water and then one of the following composition: aloes, Castile soap, āā gr. 75 ad gr. 150; boiling water, ℥iij. In the same proportions of the vehicle he experimented with colocynth, gamboge, rhubarb, jalap, scammony, etc. etc.; but the definitive result was much in favor of the aloes, as being the only drug that could be continued for several days together without inducing severe pain in the rectum and anus. Even the aloes itself has at last to be discontinued, in consequence of the irritation and tenesmus it produces. In very sensitive persons an injection may be given every other night for a week or a fortnight; while in the less susceptible, the injection may be employed every night—bedtime being the time always chosen. If the remedy is likely to prove favorable, the discharge is at once found to be daily diminishing. In some it diminishes by a half within twenty-four hours, and in others all traces have disappeared after from four to six days; while in a few, although improvement takes place, the cure is not complete. The most striking cases are those in which the uterine catarrh, after being treated by the most varied means with very indifferent success, completely disappears after five or six days' employment of the enemata. When, however, any traces of inflammation, much congestion, or exaggerated uterine sensibility, has been present, the aloes enemata have not only entirely failed, but they have notably aggravated the case. Neverthless, when the congestion or irritation of the uterus has been dependent upon some morbid processes in its vicinity, then the enemata exerts still a powerful influence over the catarrh. It is remarkable that both the aloes and some other substances, strongly purgative when taken by the mouth, exert comparatively but little effect of this kind when given as an enemata. Administered at night, they are retained until morning, and sometimes for twenty-four or even forty-eight hours; and the stools they do induce are not attended with much pain or irritation.

Although these enemata afford a certain amount of relief in the other vaginal discharges, M. Aran is not aware that a single case of vulvo-vaginal or vaginal discharge has been cured by them, however perseveringly they may have been employed, their revulsive action being strictly confined to uterine catarrh.—*Medical Times and Gazette*, Sept. 18, 1858.

2. ON THE PREVENTION OF LACERATION OF THE PERINÆUM. By *Mattei*. (*Vierteljahrsschrift, f. p. Heilk.*, 1858.)—Dr. Mattei gives the following views on the means of preventing laceration of the perinæum. It is especially necessary that the head pass the vulva in a favorable direction. This can only happen when it passes with the necessary degree of flexion. While the occiput passes under the pubic arch, the face has not yet quitted the pelvic outlet; first when the upper part of the neck comes under the pubic arch, can the extension of the head (or the separation of the chin from the breast) begin. If the distension of the perinæum begins too early, the head must pass the vulva with unfavorable diameters—namely, with the great oblique, or great or straight diagonal diameters. Such a passage easily causes laceration. Hence it is the task of the physician to prevent a premature distension by the head. This he effects by placing two fingers between the labia, or in some cases between the pubic arch and occiput, so as to bring the head downward and outward, at the same time laying the other hand on the hinder part of the perinæum, upon which the face is lying, and pushes this upward. This manœuvre is to be executed during the pains, which will thus protrude the head forward in the requisite arc. A very simple means of expediting the birth of the head consists in compressing firmly the distended perinæum with the whole hand. This resembles the squeezing out of the kernel from a cherry. On the passage of the shoulders care must also be taken lest the two shoulders pass together.—*British and Foreign Medico-Chirurgical Review*, October, 1858.

3. ECLAMPSIA AT THE EIGHTH MONTH; RECOVERY; DELIVERY OF TWO CHILDREN UNITED BY THEIR SIDES. By *Dr. Boursier*, of Creit. (*L'Union Médicale*, June, 1858.)—The case of eclampsia and double-birth of Dr. Boursier is especially interesting. A primipara, subject to hysterical attacks, was at the eighth month of gestation. A month ago she had complained of headache, for which she had been bled. Eight days before this she had been seized with considerable œdema of legs and hands. It was remarked also, that her eyes were very bright. At time of observation, eclampsia had come on; she had had five severe attacks, with coma. Largely bled, and delivery attempted. A foot was felt presenting; this was seized, and also a second; they were right and left; but after an hour and a half of extractive effort, the pelvis could not be brought down. Dr. Boursier now felt a soft body situated at the junction of the two legs. After further violent exertion, he brought the two thighs out of the vulva, and a portion of the soft, smooth, blackish mass he had before felt. He then perceived a third leg behind. An extensive adhesion between the two fœtuses was now found. The fourth leg was freed; the two trunks descended, as well as the black mass, the size of a hen's egg, which had been torn, and gave exit to some intestinal coils. During this the fits increased. The delivery was accomplished by passing a loop of a napkin over the chest of the most forward child. By dragging strongly on this backward, the head was delivered; and lastly, the second head was born. Several attacks of convulsions occurred afterwards; but she recovered favorably.

*The anatomical relation of the Fœtuses.*—Both boys, of middle size, well formed in all the non-adherent parts; they are slightly inclined toward each other. The adhesion is formed in the soft and bony parts of the abdomen, so as to constitute only one abdominal cavity. In the central part of the common belly the walls are formed by the expansion of the umbilical cord, which has the form of a funnel, whose upper part, containing the vessels, is fixed to the sternal union; below, this membrane is turned backward, and is inserted on the iliac crests. This funnel has the size of a hen's egg, and contains a large part of the intestinal organs. The cord is single, very large, and has only one vein. The chests are normal, and contain four lungs. The hearts are large, and placed forward on a level with the sternums. There are two stomachs, small, each containing a spoonful of yellowish mucosity. A spleen is attached to each. There are four kidneys and two bladders. The consequences of the fusion are two diaphragms united by their corresponding sides, preserving their points of attachment to the ribs. They possess a single liver, evidently composed by the union of two organs, for there are two gall-bladders. It is large, and occupies the central part of the umbilical pouch. The greatest anomaly consists in the atrophy of the intestine of the left child, which is reduced to the form of a cord (hollow.) The rectum, colons, cæcum, small intestine, are very distinct. This intestine has no communication with the stomach. The intestine of the right child would have acted for both. The left one would only have had the trouble of digestion.—*Ibid.*

